# DYNC1I1 Promotes the Proliferation and Migration of Gastric Cancer by Up-Regulating IL-6 Expression

**DOI:** 10.3389/fonc.2019.00491

**Published:** 2019-06-12

**Authors:** Li-Bao Gong, Ti Wen, Zhi Li, Xing Xin, Xiao-Fang Che, Jin Wang, Yun-Peng Liu, Xiu-Juan Qu

**Affiliations:** ^1^Department of Medical Oncology, the First Hospital of China Medical University, Shenyang, China; ^2^Key Laboratory of Anticancer Drugs and Biotherapy of Liaoning Province, the First Hospital of China Medical University, Shenyang, China

**Keywords:** DYNC1I1, gastric cancer, proliferation, migration, IL-6, NF-κB nuclear translocation

## Abstract

Gastric cancer is one of the top five malignant tumors worldwide. At present, the molecular mechanisms of gastric cancer progression are still not completely clear. Cytoplasmic dynein regulates intracellular transport and mitotic spindle localization, and its abnormal function is crucial for tumorigenesis, promotes tumor cell cycle progression, and tumor migration. DYNC1I1 is an important binding subunit of cytoplasmic dynein. However, studies on DYNC1I1 in tumors are currently limited. In the current study, we found that high DYNC1I1 expression in gastric cancer is associated with poor prognosis and is an independent prognostic factor. DYNC1I1 promoted the proliferation and migration of gastric cancer cells both *in vitro* and *in vivo*. DYNC1I1 also upregulated IL-6 expression by increasing NF-κB nuclear translocation. Collectively, these data revealed an important role for the DYNC1I1-driven IL-6/STAT pathway in gastric cancer proliferation and migration, suggesting that DYNC1I1 may be a potential therapeutic target for gastric cancer.

## Introduction

Gastric cancer (GC) is one of the top five malignant tumors in the world and a major cause of cancer-related death (1). Most GC cases are diagnosed at advanced stages with a 5-year survival rate of only 5% (2). Molecular therapies targeting gastric cancer have been developed, including trastuzumab that targets HER2 (3) and ramucirumab that targets VEGF-R2 (4), but the overall survival time is still only 10–12 months. Therefore, understanding the molecular mechanisms that drive the development of gastric cancer is critical to the development of new targeted therapies for this.

Cytoplasmic dynein includes cytoplasmic dynein 1 and cytoplasmic dynein 2. Cytoplasmic dynein 1 is widely expressed. In this context, cytoplasmic dynein aims at Cytoplasmic dynein 1. Cytoplasmic dynein is a large protein complex consisting of heavy chain (DYNC1H1), intermediate chain (DYNC1I1), light intermediate chain (DYNLIC), and light chain (DYNLC) that travels along the microtubules (MTs) to their minus end (5, 6). The cytoplasmic dynein core is formed by a dimer of two heavy chains. Cytoplasmic dynein has a wide range of cellular functions, including cargo transport (including mRNA, vesicles, growth factors, and transcription factors) from the cytoplasm to the nucleus and mitotic spindle localization.

There are several reports of the role of cytoplasmic dynein in cancer (7). First of all, as a regulator of precise mitosis, if its function is impaired, it may lead to abnormal mitotic process, which may alleviate tumor formation and progression, but this process may also cooperate with certain cancer-related processes to promote tumor formation (8). For example, in colorectal and cervical cancer, cytoplasmic dynein regulates tumor cell cycle progression to promote tumor progression (9). On the other hand, the current report shows than, it can interact with microtubule-associated proteins to promote 3D migration in fibrosarcoma (10). Recent research proves that cytoplasmic dynein has been found to be an important anti-tumor drug target. Cytoplasmic dynein inhibitors have been developed, including the aminothiazole dynarrestin, which blocks dynein activity thereby suppressing the Hedgehog pathway downstream of Smoothened (11). Cytoplasmic dynein can also be inhibited by the tumor suppressor REIC/DKK-3 (12).

DYNC1I1 is an important cargo binding subunit of cytoplasmic dynein. It determines the specificity of the combined cargos. Previous studies have shown that loss of DYNC1I1 could result in split hand/split foot malformation type I (13). The role of DYNC1I1 in cancer is controversial. The latest research shows that DYNC1I1 is significantly downregulated in patients with glioblastoma (GBM), and low DYNC1I1 expression is related to poor patient survival (6). Previous studies have shown that in colorectal cancer, the intestinal cancer inhibitor Calgranulin B binds to various proteins, including DYNC1I1, to inhibit tumor cell proliferation (14). However, the sub-chromosomal region located in 7q21.3 was found to be amplified in hepatocellular carcinoma, and DYNC1I1 may be a proto-oncogene located at this site (15). High expression of the cytoplasmic dynein major subunit DYNC1H1 was also found in drug-resistant gastric cancer cells (16). DYNC1I1, as the important binding subunit of cytoplasmic dynein, the function of it in gastric cancer needs to be further explored.

This study demonstrates for the first time that DYNC1I1 is independently associated with poor prognosis in gastric cancer. DYNC1I1 promoted the proliferation and migration of gastric cancer cells *in vitro* and *in vivo*. These effects were achieved through the upregulation of IL-6 expression. Blocking the IL-6/STAT3 axis abolished the effects of DYNC1I1 on gastric cancer cells. DYNC1I1-mediated upregulation of IL-6 expression resulted from increased nuclear translocation of the P65 (a member of the NF-κB family). These results indicate that DYNC1I1 promotes proliferation and migration and is a potential therapeutic target for patients with gastric cancer.

## Materials and Methods

### Cell Lines and Culture Condition

HGC-27, SGC-7901, and MGC-803 gastric cancer cells were routinely cultured under the conditions of RPMI1640 (Gibco, Carlsbad, CA, USA) with 10% fetal bovine serum, 100 U/ml penicillin and 100 ug/ml streptomycin at 37°C, 5%CO2. Experiments were carried out using logarithmic growth phase cells.

### Bioinformatics Analysis

GSE62254 is downloaded from the GEO database (https://www.ncbi.nlm.nih.gov/geo). Kaplan Meier plotter (http://kmplot.com/analysis/index.php?p=service&cancer=gastric) was used to map Kaplan-Meier survival curves for gastric cancer patients with different DYNC1I1 expression levels. Download the gene positively associated with DYNC1I1 in the gastric cancer dataset on cBioPortal for Cancer Genomics, a list of 1,753 genes with the highest co-expression correlation (Spearman score> 0.3) was submitted to DAVID Bioinformatics Resources 6.7 (http://david.abcc.ncifcrf.gov) for gene pathway enrichment analysis (17).

### MTT Assay

The cell suspension containing HGC-27 was added to a 96-well plate at a concentration of 2 × 10^5^/mL and containing SGC-7901 cell suspension at a concentration of 5 × 10^4^/mL. MGC-803 cell suspension at a concentration of 2 × 10^4^/mL. The experiment was set as a blank group, a control group, and an experimental group that knocked down DYNC1I1 or over experissed DYNC1I1. The blank group is a cell-free medium alone. Set 4 sub holes in each group. Different treatment factors are applied after the cells are attached to the wall. The control group was knocking down the useless sequence. The experimental group was knocking down DYNC1I1 or over experising DYNC1I1. After the specified incubation time (0, 24, 48, 72, 96 h), 20 uL of MTT solution (5 mg/mL) was added to each well, and the mixture was gently shaken and incubated at 37°C and 5% CO 2 for 4 h. Aspirate each well with a vacuum aspirator and add 200 uL of dimethyl sulfoxide (DMSO) solution. The 96-well plate was shaken for 10 min on a horizontal shaker. The OD value at 570 nm was measured using a microplate reader and calculated according to the formula value. Cell viability: relative cell activity = (OD570 measurement—OD570 blank)/(OD570 control—OD570 blank) × 100%.

### Protein Extraction and Western Blot Analysis

The control and treatment groups were separately collected by lysis buffer, lysed at 4°C for 40 min, centrifuged at 13,000 rpm for 25 min, take the supernatant and the Coomassie Brilliant Blue method was used for protein quantification. Mix with 3 × Loading Buffer and boil at 95°C for 5 min. The sample was subjected to electrophoresis in an 8% SDS-polypropylene gel at a concentration of 30–50 ug/lane for 3 h and then transferred to a nitrocellulose membrane. After blocking with 5% skim milk for 1 h, the transfer membrane was cut according to the molecular weight of the pre-stained marker, and DYNC1I1 antibody (Abcam 23905 1:1,000), Actin antibody (Santa sc-47778 1:1,000), STAT3 antibody (CST 4904S 1:2,000), p-STAT3 antibody (CST 9131 1:500) were added. Four degree Celsius overnight. Wash TTBS 4 times for 10 min each time, then add horseradish peroxide-labeled goat anti-mouse (1:2,000) or goat anti-rabbit (1:2,000) secondary antibody to room temperature for 30 min, and again wash the membrane 4 times with TTBS, using the ECL method to visualize.

### Colony Formation Assay

To test the long-term effects of DYNC1I1 on the proliferation of gastric cancer cells, we inoculated SGC-7901 (500 cells/well) and HGC-27 (800 cells/well) transfected with sh-DYNC1I1-1 and sh-DYNC1I1-2 and MGC-803 (500 cells/well) with overexpression-DYNC1I1 into 12-well culture plates. During the week, the colony growth was observed with an inverted microscope. After 2 weeks, the cells were fixed with methanol, stained with 0.5% crystal violet, and the colonies number were counted using Image-Pro Plus.

### Cell Transfection

The gastric cancer cells were inoculated into a six-well plate at 1.5 × 10^5^/well, after 24 h, siRNA/shRNA transfection was then carried out with Lipofectamine 2000 (Invitrogen, Carlsbad, CA, USA) according to the manufacturer's instructions. Continue to process cells at different time points according to different needs of the experiment, including RNA isolation, colony formation assay were further performed, respectively.

### Migration Assay

HGC-27, SGC-7901 and MGC-803 cells were seeded and transfected as described above. 24 h after the transfection, cells were trypsinized, suspended in culture medium without FBS, SGC-7901 cells count 4 × 10^4^ cells, HGC-27 cells count 2.4 × 10^4^ cells, MGC-803 cells count 4 × 10^4^ cells, and 200 uL cell suspension is prepared by using culture medium without FBS, and fully mixed. Five hundred microliter of 2.5% FBS medium was added to the lower chamber of Transwell, and the mixed cell suspension was placed in the upper chamber of an 8 μm microporous filter and incubated in a 37°C incubator for 24 h. For interleukin-6 (IL-6) or IL-6 antibody treatment, cells were cultured in medium containing 200 ng/mL IL-6 (R&D Systems, MN, USA) or 400 ug/ml IL-6 neutralizing antibody(R&D Systems, MN, USA). After removing the upper chamber, gently wipe off the unmigrated cells in the upper chamber with a cotton swab, dry at room temperature for 10 min, fix with 4% paraformaldehyde for 1 min, stain with Reiter for 1 min, and then mix and dilute Giemsa for 40 min. Take a photo with a microscope. Five fields of view were randomly selected under a 200 × high power microscope, and the average number of transmembrane cells was counted and counted as the number of migrated cells in the chamber.

### Total RNA Extraction and Real-Time PCR

Total cellular RNA was extracted using Trizol and the procedure was performed according to the Trizol reagent instructions, all operations are done on ice. RNA was extracted and reversely transcribed into cDNA according to the PrimeScript® RT reagent Kit with gDNA Eraser (Takara, Japan) kit protocol. The SYBR® Premix Ex TaqTM II method detects mRNA relative expression. The GAPDH was used as the internal reference. Corresponding primer sequences:DYNC1I1 Primer:DYNC1I1(sense 5′-CGG AAG GAA GAG GAG AG GA-3′, antisense 5′-GGA GGG AGA CAT AGG GG TT-3′), IL-6(sense 5′-TCT CCA CAA GCG CCT TCG-3′, antisense 5′-CTC AGG GCT GAG ATG CCG-3′), GAPDHs(sense 5′-CAC CCA CTC CTC CAC CTT TG-3′, antisense 5′-CCA CCA CCC TGT TGC TGT AG-3′), the relative mRNA expression of DYNC1I1, and IL-6 were estimated by ΔΔCt and normalized to GAPDH.

### Immunoprecipitation

Thirty five microliter Protein G-sepharose beads (DYNC1I1) and 35 ul Protein A-sepharose beads(P65) were added to the EP tube and the beads were washed twice with PBS and lysis buffer, respectively. The total protein of the control and treatment groups was extracted with lysis buffer, applying Coomassie Bright. The total protein concentration was quantitatively extracted by the method. Forty microliter of protein was taken from each sample as input, and 3 × Buffer was added, after mixing boiled at 95°C for 5 min. The remaining protein lysate and 10 ug of anti-DYNC1I1 antibody and 10 ug of anti-P65 antibody(CST 8242S) in each sample were then added to Protein G-sepharose beads and Protein A-sepharose beads, respectively, and the solution containing beads, protein lysate, and antibody was slowly shaken overnight at 4°C. After overnight, the beads were washed four times with lysis buffer, the supernatant was aspirated, and 2 × Buffer 40–50 ul was added and boiled for 5 min, and the samples were subjected to Western blot analysis.

### ELISA

The protein level of IL-6 in the cell culture supernatant was measured by an IL-6 ELISA kit (R&D Systems, MN, USA). DYNC1I1 promotes the proliferation of gastric cancer cells, therefore, the effect of cell proliferation on IL-6 levels at different time points was eliminated by cell number.

### Animal Experiment

Cell preparation and subcutaneous tumor formation in nude mice, tumor metastasis of tail vein:SGC-7901 cells were transfected with DYNC1I1 knockdown lentivirus, and then passaged and screened. Subcutaneous tumor formation: a single cell suspension at a concentration of 2 × 10^6^/200 ul was prepared. The cells were injected into the abdomen of the BABL/C female nude mice (Beijing Vital River Laboratory Animal Counting Co, Ltd.) for 4–6 weeks for subcutaneous tumor formation. Venous lung metastasis into tumors: Cells suspension was prepared at a concentration of 1 × 10^6^/100 ul. The cells were injected into the tail vein of BABL/C female nude mice (Beijing Vital River Laboratory Animal Counting Co, Ltd.) for 4–6 weeks. Observation indicators and methods: after the nude mice were injected subcutaneously into the tumor cells, the tumor volume was measured every 2 days after the tumor was formed and the tumor was visible and stable. The volume calculation formula was: volume (mm3) = longitude × short passage^2^/2. Mice injected into the tail vein were monitored for body weight every 2 days about 5 weeks after injection into tumor cells. The mice were sacrificed by cervical dislocation about 19 days after subcutaneous injection of tumor cells in nude mice. After the tumors were exfoliated from nude mice, the tumor volume was measured and the tumor weight was weighed. The mice injected into the tail vein were sacrificed by cervical dislocation about 9 weeks after the injection of the tumor cells, and the lung tissues of the mice were detached. Pulmonary metastases were visually observed and confirmed by HE.

### Immunohistochemistry

After subcutaneous tumors of the mice and the lungs of the mice in which the tumor cells were injected into the tail vein were embedded and sliced as required, set the oven to a temperature of 65°C and bake at a constant temperature for 120 min. Performing according to immunohistochemistry kit. The staining was evaluated by scanning the entire tissue specimen under low magnification (×10) and confirmed under high magnification (×20 and × 40). The protein expression was visualized and classified based on the percentage of positive cells and the intensity of staining. The heterogeneity of staining was scored as 0 (≤5%), 1 (6–25%), 2 (26–50%), or 3 (>51%). After making the calculations, we evaluate DYNC1I1 expression by determining the staining index with scores of 0, 1, 2, 3, 4, 6, or 9. Negative immunohistochemical expression is defined as the index ≤3 and positive expression was considered if the index was >4. Final scores were assigned by two independent pathologists.

### Patients and Tissue Samples

A total of 30 GC and paired adjacent non-cancerous gastric FFPE tissue (not <20 mm away from GC) specimens surgically resected primary gastric cancer patient specimens were obtained from the First Hospital of China Medical University between Jan 1st 2017 and Dec 31st 2018. Age, sex and pTNM stage were evaluated following medical charts and pathology records. pTNM stage was examined according to the 8th edition of AJCC cancer staging manual. No patients had received any neoadjuvant chemotherapy and radiotherapy. All research involving human participants were approved by the Ethics Committee of China Medical University. Written informed consents were obtained from all the participants in accordance with the Helsinki Declaration.

### Statistical Analysis

SPSS version 22.0 is used for statistical analysis. The χ2 test was used to examine the possible association between DYNC1I1 expression and clinicopathological factors. The Kaplan-Meier method was used to estimate the probability of patient survival. The Cox regression model was used for multivariate analysis. Differences between groups were compared by using Student's *t*-test. Each experiment was repeated three times and the data was expressed as mean + standard deviation. And P <0.05 was considered to be statistically significant.

## Results

### High DYNC1I1 Expression Predicts Poor Prognosis in Patients With Gastric Cancer

**Figure 1:**
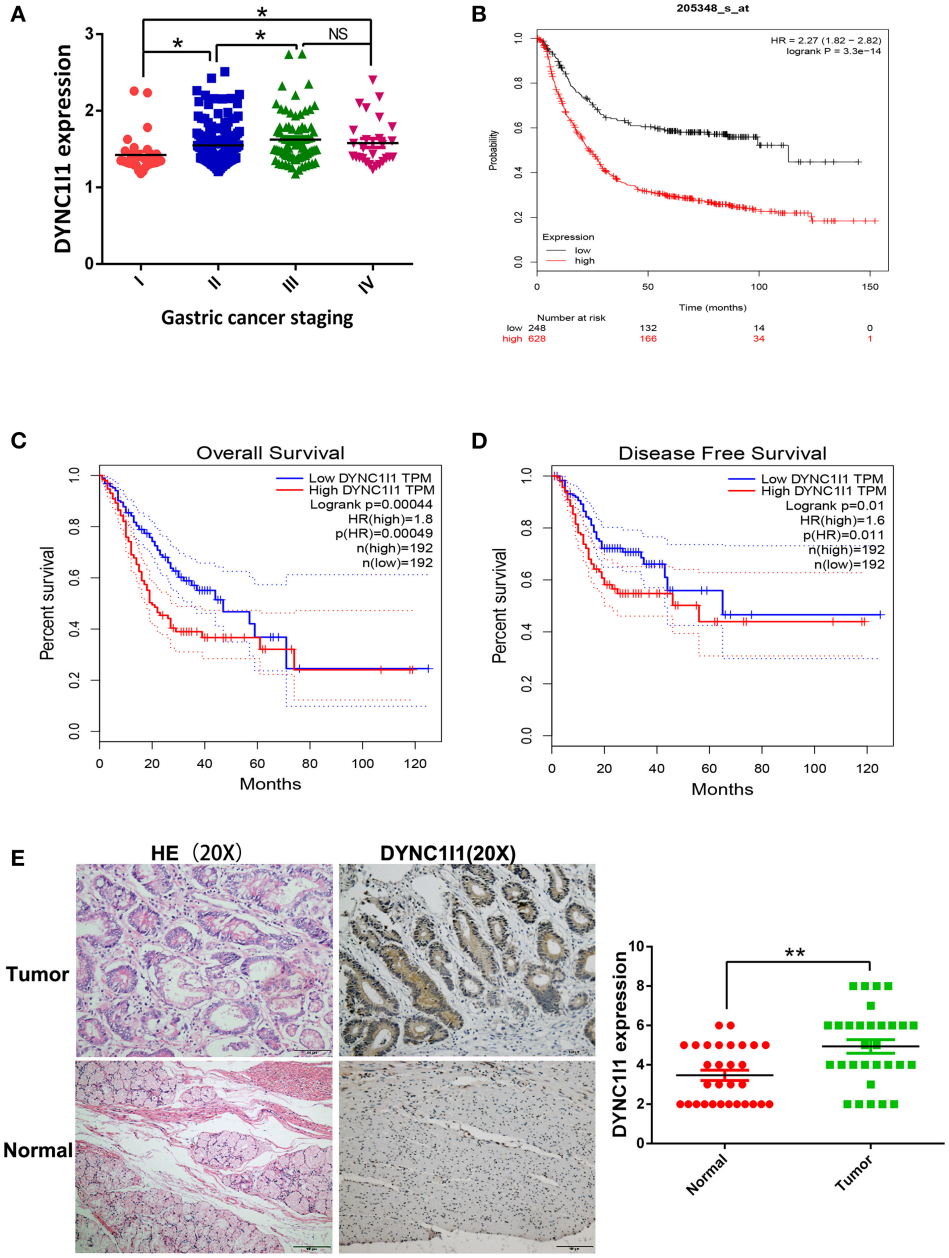
High DYNC1I1 expression predicts poor prognosis in patients with gastric cancer. **(A)** Analysis of the relationship between the expression of DYNC1I1 and the staging of gastric cancer by GSE62254 gastric cancer dataset. **(B)** Kmplot analysis of DYNC1I1 expression and survival of gastric cancer patients. **(C)** COX analysis of DYNC1I1 and gastric cancer OS on the GEPIA website. **(D)** COX analysis of DYNC1I1 and gastric cancer DDFS on the GEPIA website. **(E)** The expression levels of DYNC1I1 in 30 pairs of GC tissues and the adjacent gastric cancer tissue, measured by IHC. The left were the HE stained sections, magnification × 100; and IHC-stained sections, magnification × 100; (^*^P < 0.05, ^**^P < 0.01).

To study the role of DYNC1I1 in gastric cancer, GSE62254 data was used to investigate the clinical value of DYNC1I1 in gastric cancer. The data were divided into high DYNC1I1 expression and low DYNC1I1 expression groups according to DYNC1I1 gene expression. The chi-square test was used to determine the relationships between DYNC1I1 expression and the clinicopathological features of gastric cancer. This analysis showed that DYNC1I1 expression was associated with the depth of invasion (*P* = 0.003), lymph node status (*P* = 0.001), and TNM stage (*P* = 0.032) (Table 1). As shown in Table 2, the T stage (HR = 0.385, 95% CI = 0.274–0.541, *P* = 0.000), N stage (HR = 2.966, 95% CI = 2.093–4.202, *P* = 0.000), TNM Stage (HR = 3.847, 95% CI = 2.729–5.422, *P* = 0.000), and DYNC1I1 expression levels (HR = 2.227, 95% CI = 1.567–3.165, *P* = 0.000) were prognostic risk factors based on univariate analysis. In addition, multivariate analysis showed that T stage (HR = 1.854, 95% CI = 1.289–2.642, *P* = 0.001), *N* stage (HR = 2.087, 95% CI = 1.444–3.017, *P* = 0), TNM stage (HR = 2.352, 95% CI = 1.343–4.121, *P* = 0.003), and DYNC1I1 expression (HR = 2.095, 95% CI = 1.450–3.026, *P* = 0) were independent prognostic risk factors (Table 2). As shown in Figure 1A, the level of DYNC1I1 in gastric cancer increased with the progression of the disease. DYNC1I1 expression in stage II tumors was significantly elevated compared to stage I tumors (*P* = 0.0118), and the DYNC1I1 expression further increased in stage III and IV tumors. To further explore the prognostic value of DYNC1I1 expression in gastric cancer, we analyzed the overall survival (OS) of gastric cancer patients based on the level of DYNC1I1 expression and found that high DYNC1I1 expression was associated with a shorter OS (*P* < 0.001) (Figure 1B). Multivariate Cox analysis revealed that DYNC1I1 was an independent prognostic indicator for gastric cancer (*P* < 0.05) (Figures 1C,D). Then DYNC1I1 expressions were detected using immunohistochemical analysis. The relative DYNC1I1 expression level was significantly increased in GC tumors compared to the paired normal tissue (*p* < 0.01, Figure 1E). Patient details can be found in Supplementary Table 1. At the same time, to determine differences of DYNC1I1 mRNA expression in tumor and normal tissues, the DYNC1I1 mRNA levels in GC tumors and normal tissues were analyzed using the Oncomine database. This analysis revealed that the DYNC1I1 expression was higher in GC tumors compared to the normal tissues (fold change = 1.075, *p*-value < 0.01) (Supplementary Figure 1). Together, these data indicated that DYNC1I1 expression increased with the progression of gastric cancer stage, and high DYNC1I1 expression was associated with the worse outcomes in gastric cancer.

**Table 1:** DYNC1I1 expression and clinicopathological characteristics in 298 gastric cancer.

		**DYNC1I1 expression**		
**Clinicopathological characteristics**	**Cases (*n* = 298)**	**High (%)**	**Low (%)**	***P*-value**
**SEX**				
Male	197	90 (45.7)	107 (54.3)	0.055
Female	101	58 (57.4)	43 (42.6)	
**AGE (YEARS)**				
≤60	117	60 (51.3)	57 (48.7)	0.653
>60	181	88 (48.6)	93 (51.4)	
**TNM STAGE**				
I–II	193	87 (45.1)	106 (55.0)	0.032
III–IV	105	61 (58.1)	44 (42.0)	
**LOCAL INVASION**				
T1–T2	186	80 (43.0)	106 (57.0)	0.003
T3–T4	112	68 (60.7)	44 (39.3)	
**LYMPH-NODE METASTASIS**				
0–2	168	69 (41.1)	99 (59.0)	0.001
>2	130	79 (60.8)	51 (39.2)	

**Table 2:** Univariate and multivariate OS Analysis in GSE62254 dataset.

**Variables**	**Univariate analysis**			**Multivariate analysis**		
	**HR**	**95%CI**	***P*-value**	**HR**	**95%CI**	***P*-value**
DYNC1I1 expression	2.227	1.567–3.165	0	2.095	1.450–3.026	0
Age (years) (<60 vs. ≥60)	1.162	0.821–1.646	0.397	0.858	0.720–1.023	0.088
Gender (Male vs. Female)	0.833	0.587–1.184	0.309	1.053	0.875–1.267	0.584
T stage	0.385	0.274–0.541	0	1.854	1.289–2.642	0.001
N stage	2.966	2.093–4.202	0	2.087	1.444–3.017	0
TNM stage	3.847	2.729–5.422	0	2.352	1.343–4.121	0.003

### DYNC1I1 Promotes Cell Growth and Migration of Gastric Cancer Cells *in vitro*

**Figure 2:**
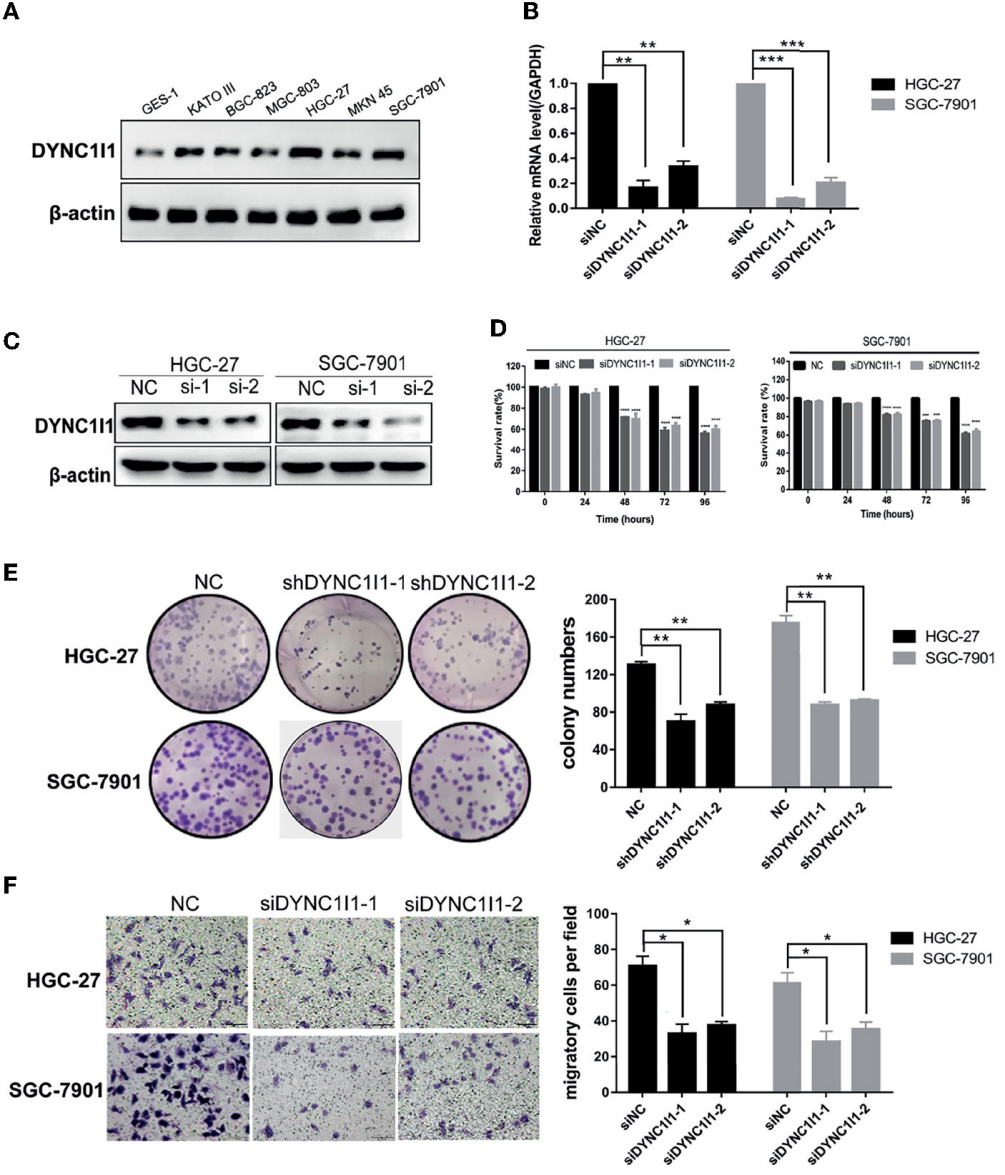
Knockdown of DYNC1I1 leads to suppression of gastric cancer progression and migration *in vitro*. **(A)** Western blot shows DYNC1I1 protein expression levels in normal gastric cells and different gastric cancer cells. **(B)** and **(C)** RT-qPCR and western blot show DYNC1I1 transcription level and protein expression after transient knockdown DYNC1I1 gene by using siRNAs for 48 h. **(D)** MTT shows cell viability of gastric cancer cells after knocking down DYNC1I1 for 0, 24, 48, 72, 96 h. **(E)** Colony formation shows gastric cancer cells form colony ability after knocking down DYNC1I1. **(F)** Transwell assay displays the change in migration of gastric cancer cells after knocking down DYNC1I1 or not (magnification ×200). (^*^P < 0.05, ^**^P < 0.01,^***^P < 0.001,^****^P < 0.0001, *n* = 3, student *t*-test, means ± 95% CI).

**Figure 3:**
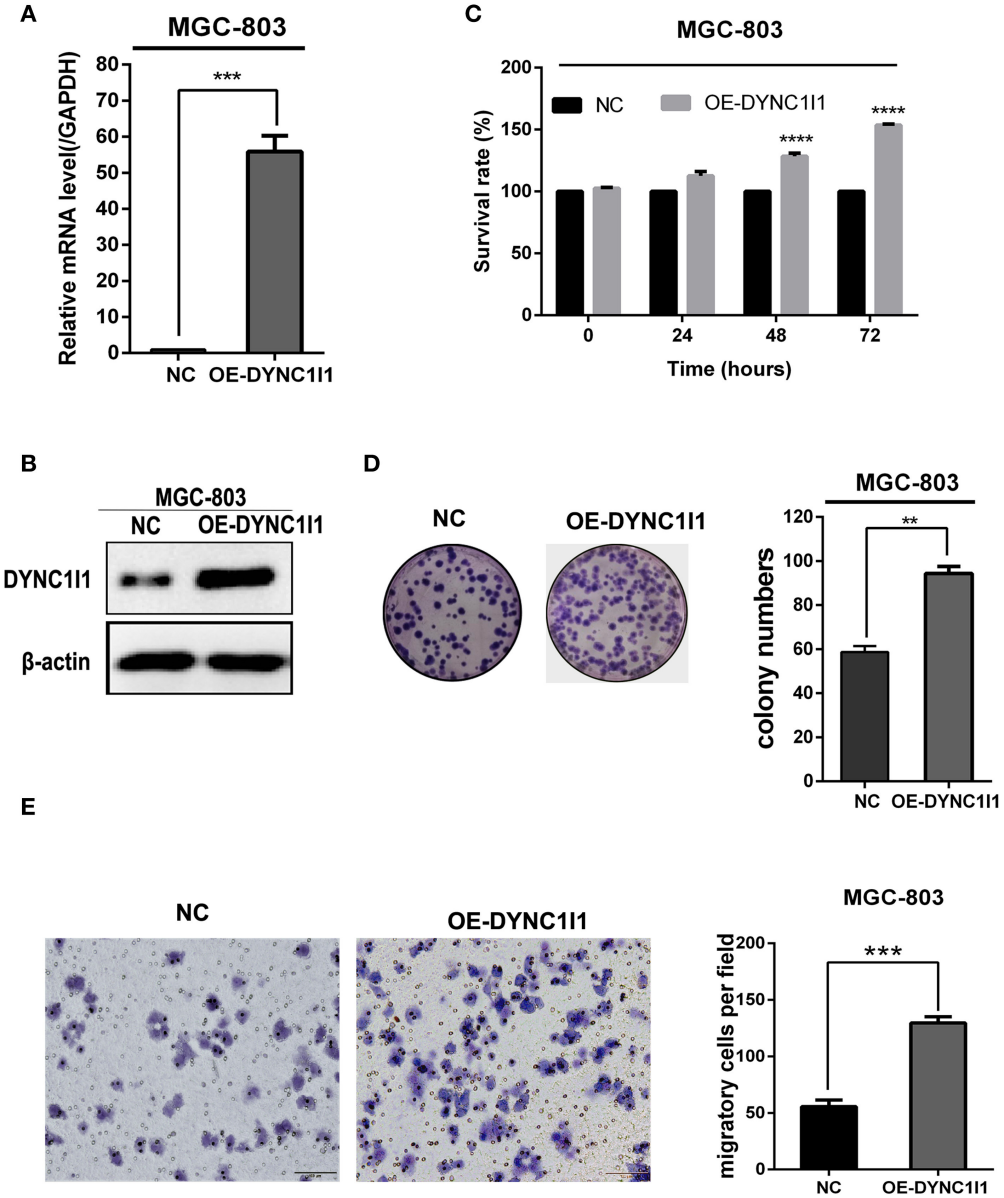
Overexpression of DYNC1I1 promotes cell growth and migration of gastric cancer cells *in vitro*. **(A,B)** RT-qPCR and Western blot were used to detect DYNC1I1 expression in MGC-803. **(C)** MTT shows cell viability of gastric cancer cells after overexpressing DYNC1I1 for 0, 24, 48, 72, 96 h. **(D)** Transwell migration assay of MGC-803 with DYNC1I1 overexpression. Original magnification, x200. Scale bar: 100 μM. β-actin was used as a loading control in Western blot. (^**^P < 0.01, ^***^P < 0.001, ^****^P < 0.0001, *n* = 3, student *t*-test, means ± 95% CI).

To understand the biological effects of DYNC1I1 in gastric cancer cells, DYNC1I1 protein levels in six gastric cancer cell lines were measured by Western blot. DYNC1I1 was expressed to different degrees in these cell lines, and greater expression was observed in the gastric cancer cells compared to normal gastric cells GES-1 (Figure 2A). RT-qPCR and Western blot were used to verify the transient knockdown of DYNC1I1 mRNA and protein knockdown efficiency in gastric cancer cells, respectively (Figures 2B,C). Then MTT and colony formation assays were used to assess the effect of DYNC1I1 knockdown on cell proliferation (Figures 2D,E). By 48–96 h after DYNC1I1 knockdown in HGC-27 cells, proliferation decreased to ~20–40% of that observed without DYNC1I1 knockdown (*P* < 0.05). Similar results were obtained with SGC-7901 cells. Consistent with the MTT results, knockdown of DYNC1I1 levels resulted in a 50% reduction in the number of colonies formed by HGC-27 and SGC-7901 cells (Figure 2E). In addition, knockdown of DYNC1I1 decreased the migration ability of both HGC-27 and SGC-7901 by 50% (*P* < 0.05) compared to negative control cells (Figure 2F). This decrease was observed 48 h after DYNC1I1 knockdown. At the same time, proliferation was only reduced by about 20%. These results indicated that the differences in migration were not due to differences in the rate of proliferation. For further analyses, overexpression of DYNC1I1 in the MGC-803 cell line, in which DYNC1I1 was relatively low in expression, and overexpression efficiency were detected by RT-qPCR and Western blot, respectively (Figures 3A,B). The MTT assay indicated that overexpression of DYNC1I1 in MGC-803 cells enhanced the proliferation of MGC-803 cells in a time-dependent manner (Figure 3C), by 48–72 h after DYNC1I1 overexpression in MGC-803 cells, proliferation increased to ~20–50% of that observed without DYNC1I1 overexpression (*P* < 0.05). Similarly, colony formation experiments have demonstrated that overexpression of DYNC1I1 can promote long-term proliferation of gastric cancer cells (Figure 3D). Furthermore, Transwell assays indicated that the migration capacity of MGC-803 cells can be significantly enhanced after overexpression of DYNC1I1 (Figure 3E). Overall, the *in vitro* experiments demonstrated that knockdown of DYNC1I1 suppressed cell growth and migration of gastric cancer cells.

### DYNC1I1 Leads to Promotion of Gastric Cancer Progression and Migration *in vivo*

**Figure 4:**
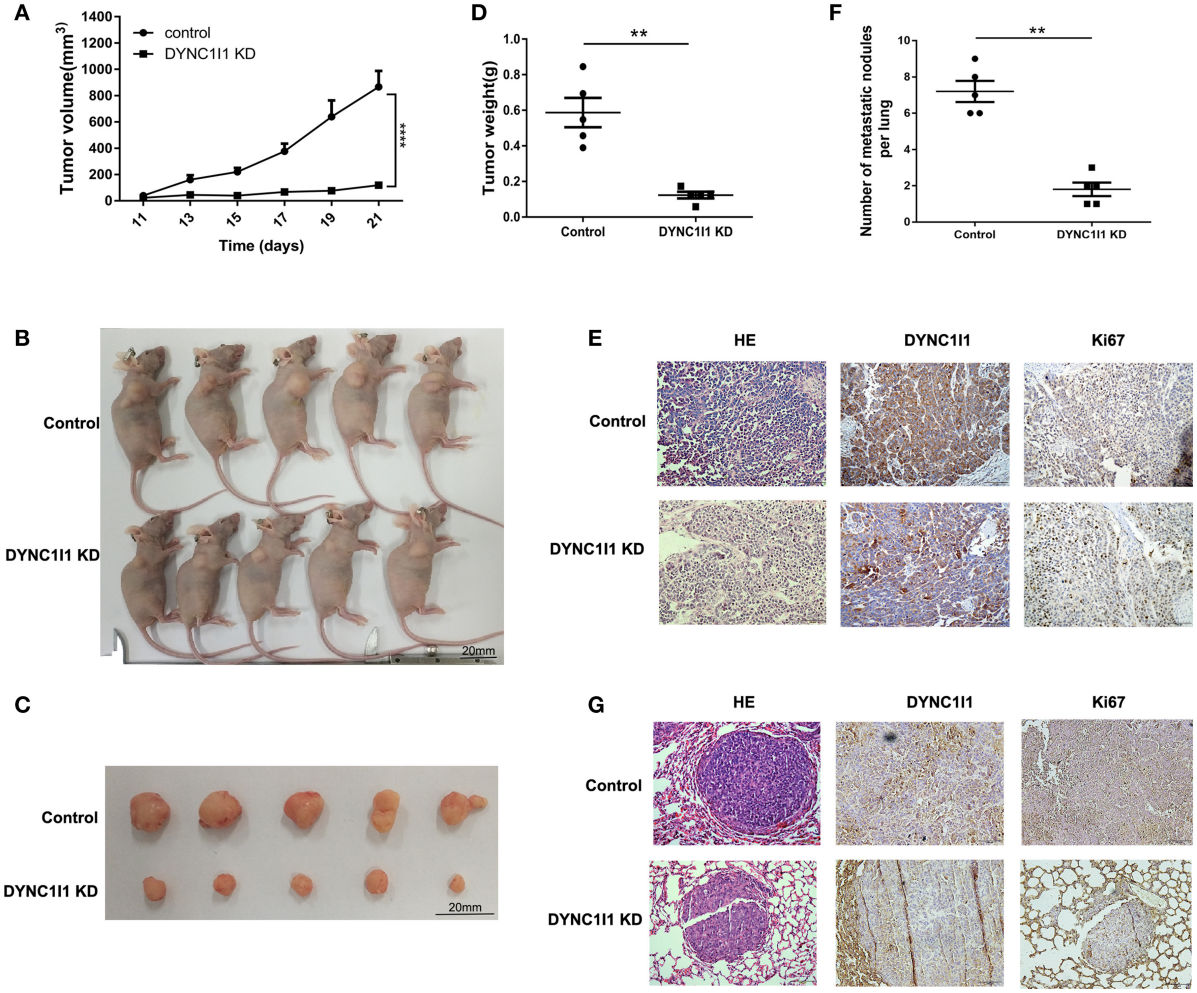
DYNC1I1 leads to promotion of gastric cancer progression and migration *in vivo*. **(A)** *In vivo* growth curve of xenografts formed by subcutaneous injection of SGC-7901 cells stably transfected with NC, KD DYNC1I1 into the dorsal flanks of nude mice (*n* = 5 for each group). **(B)** and **(C)** Representative images. **(D)** Tumor weight. **(E)** HE and representative immunohistochemistry (IHC) staining with DYNC1I1 and Ki67 in subcutaneous tumor tissue. **(F)** Lung metastatic colonization of nude mice treated with tail vein injection of SGC-7901 cells stably transfected with NC, KD DYNC1I1. **(G)** HE and representative immunohistochemistry (IHC) staining with DYNC1I1 and Ki67 in lung metastatic tumor tissue. Ki67:nuclear related antigen Ki67 (^**^P < 0.01, ^****^P < 0.0001, student *t*-test, *n* = 5 for each group).

Xenograft models were used to study the role of DYNC1I1 in the occurrence and metastasis of gastric tumors *in vivo*. As shown in Figure 4A, SGC-7901 DYNC1I1 KD cells produced smaller tumors than the control SGC-7901 NC cells. Figures 4B,C were the images of subcutaneous tumor formation. Indeed, the weights of the tumors collected 22 days after subcutaneous injection were significantly higher for the SGC-7901 NC tumors compared to the SGC-7901 DYNC1I1 KD tumors (Figure 4D). H&E staining confirmed the tumorigenic properties of the collected tumors (Figure 4E). Immunohistochemical staining verified that DYNC1I1 protein expression was downregulated in the tumors in the knockdown group. Furthermore, the levels of Ki-67 (proliferation marker) were significantly decreased in the knockdown group. We next examined the role of DYNC1I1 in tumor metastasis *in vivo* by tail vein injection of SGC-7901 DYNC1I1 KD and SGC-7901 NC cells into mice. As shown in Figures 4F,G, mice injected with SGC-7901 DYNC1I1 KD cells had significantly fewer lung metastases compared to the control group. Immunohistochemical staining showed that the levels of DYNC1I1 and Ki-67 were significantly lower in the SGC-7901 DYNC1I1 KD tumors compared to the control tumors. These data demonstrated that knockdown of DYNC1I1 suppressed tumorigenesis and metastasis of gastric cancer cells *in vivo*.

### DYNC1I1 May Function by Regulating the IL-6 Pathway

**Figure 5:**
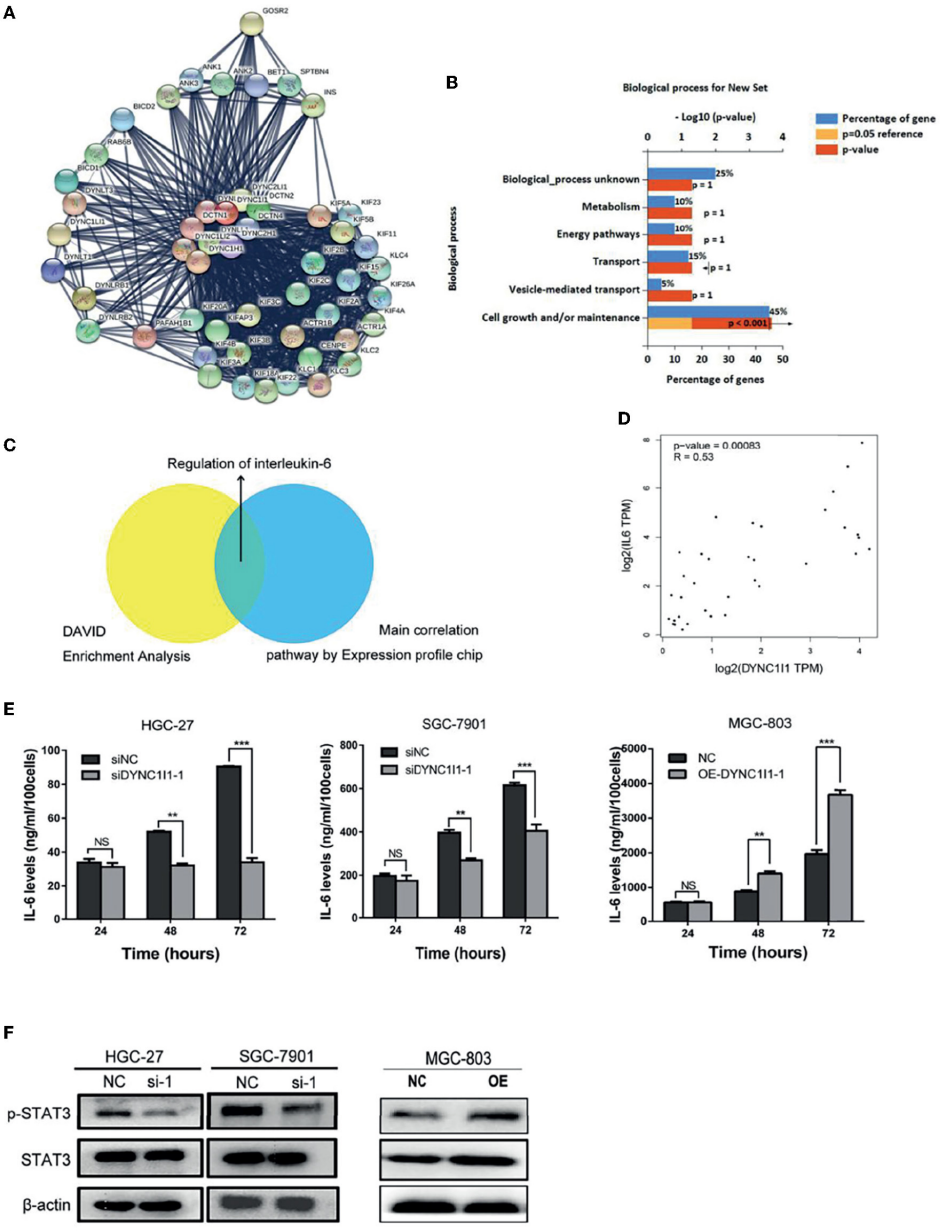
DYNC1I1 may function by regulating the IL-6 pathway. **(A)** Sting Website enrichment DYNC1I1 related genes and FunRich software enrichment related pathway. **(B)** FunRich software for Biological Pathway Enrichment Analysis. **(C)** Expression profile chip shows the main correlation pathway about DYNC1I1. **(D)** GEPIA website analysis the correlation between DYNC1I1 and IL-6 in gastric cancer. **(E)** Elisa shows the change of IL-6 expression after knocking down or overexpression DYNC1I1. **(F)** Western blot indicates the differential levels of STAT3, P-STAT3. β-actin was used as a loading control in Western blot. (^**^P < 0.01, ^***^P < 0.001, *n* = 3, student *t*-test, means ± 95% CI).

To uncover the specific mechanism by which DYNC1I1 promotes gastric cancer cell growth and migration, genes that could interact with DYNC1I1 were filtered using the STRING online database (STRING: functional protein association networks) (18) (Figure 5A). The selected genes were enriched according to biological processes using FunRich software. The analysis revealed that 52% of the related genes were involved in cell growth and maintenance (Figure 5B), which is consistent with the *in vivo* and *in vitro* results for DYNC1I1 obtained in this study. Then use the DAVID website to analyze the genes that positively related to DYNC1I1. GO analysis showed that these genes were particularly enriched in the Calcium signaling pathway, Adrenergic signaling in cardiomyocytes, IL-6-Jak-STAT signaling pathway, Insulin signaling pathway, Ubiquitin mediated proteolysis. Among them, the IL-6-Jak-STAT signaling pathway was significantly associated with growth and migration (Table 3). Affymetrix scanner microarray genome-wide expression analysis was applied after HGC-27 cells with knockdown of DYNC1I1, from the results of GO analysis of that the down-regulated genes, “Negative regulation of interleukin-6 secretion” was the top one pathway (Table 4). These results suggest that IL-6 might play a leading role in the growth and migration of gastric cancer cells mediated by DYNC1I1 (Figure 5C). Previous studies have shown that IL-6 is closely related to gastric cancer, but its correlation with DYNC1I1 has no reports yet. Thus, GEPIA Website was used to analysis the relationship between IL-6 and DYNC1I1 (Figure 5D). It was found that IL-6 and DYNC1I1 were positively correlated in gastric cancer with a correlation coefficient of 0.53 (*P* < 0.05). Furthermore, the IL-6 concentration in culture supernatants at different time points after DYNC1I1 knock down in HGC-27 and SGC-7901 cells were measured by ELISA and found that inhibition of DYNC1I1 significantly reduced IL-6 protein levels in the cell culture medium (Figure 5E). Conversely, overexpression of DYNC1I1 in MGC-803 cells increased IL-6 protein levels in the cell culture medium. Western blot showed that STAT3 phosphorylation, which is a classical IL-6 downstream target, was also significantly downregulated following the knockdown of DYNC1I1 expression, overexpression of DYNC1I1 in MGC-803 cells upregulated the expression of STAT3 phosphorylation, but the background has not changed significantly (Figure 5F). These results suggest that DYNC1I1 may regulate the biological behavior of gastric cancer cells by regulating the levels of IL-6 and that the IL-6-Jak-STAT pathway may be involved in DYNC1I1 regulation of gastric cancer cell proliferation and migration.

**Table 3:** DAVID pathway enrichment analysis.

**Term**	***P*-Value**	**Fold enrichment**
hsa04020:Calcium signaling pathway	0.002906825	1.045385779
hsa04261:Adrenergic signaling in cardiomyocytes	0.010896802	1.045385779
hsa04630:IL-6-Jak-STAT signaling pathway	0.011334596	1.045385779
hsa04910:Insulin signaling pathway	0.014915057	1.045385779
hsa04120:Ubiquitin mediated proteolysis	0.015508796	1.045385779

**Table 4:** Chip results pathway enrichment analysis.

**Term_description**	***P*-value**	**Fold enrichment**
Dichotomous subdivision of terminal units involved in salivary gland branching	0.005470757	6.64300121
Regulation of dendritic spine development	0.008956241	5.535498185
Heparan sulfate proteoglycan biosynthetic process, enzymatic modification	0.021448046	5.312258065
Toll signaling pathway	0.021448046	5.312258065
Negative regulation of interleukin-6 secretion	0.032099571	4.426612903

### DYNC1I1 Promotes Cell Growth and Migration of Gastric Cancer Cells Through IL-6

**Figure 6:**
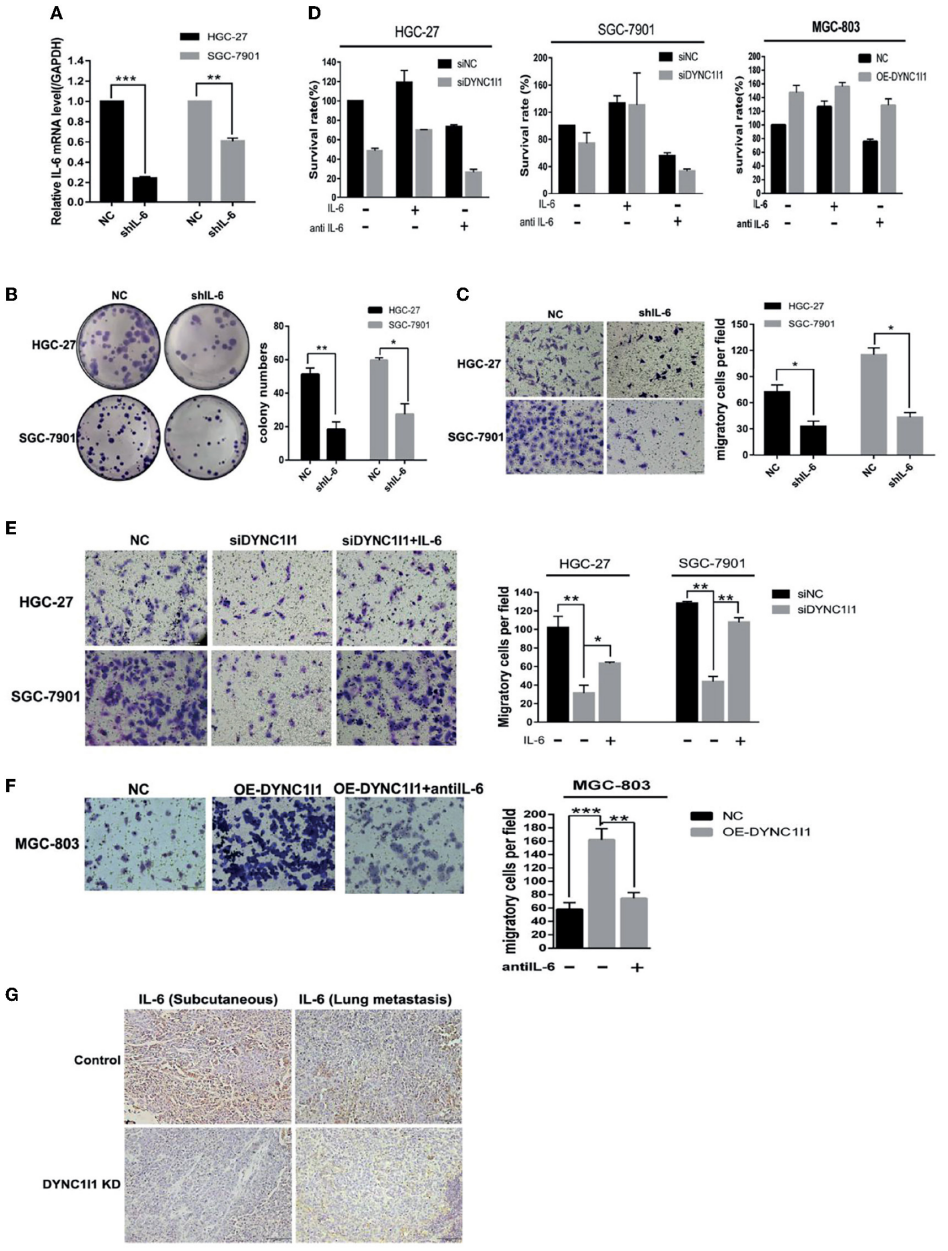
DYNC1I1 promotes proliferation and migration of gastric cancer cells through IL-6. **(A)** RT-qPCR shows IL-6 transcription levels after knockdown IL-6 gene by using shRNA for 48 h. **(B)** Colony formation shows gastric cancer cells form colony ability after knocking down IL-6. **(C)** Transwell assays displays the change in migration of gastric cancer cells after knocking down IL-6 or not. **(D)** MTT shows proliferation ability of gastric cancer cells after adding IL-6 or IL-6 neutralizing antibody after knocking down or overexpression DYNC1I1. Add IL-6 or IL-6 neutralizing antibody 48 h after knocking down DYNC1I1. **(E)** Transwell assays shows the change in migration of gastric cancer cells after adding IL-6 after knocking down DYNC1I1. **(F)** Transwell assays shows the change in migration of gastric cancer cells after adding IL-6 neutralizing antibodies after overexpression DYNC1I1. (magnification ×200) **(G)** Immunohistochemical detection of IL-6 expression in subcutaneous and lung metastatic tumors of mice tumor tissue(magnification ×100). (^*^P < 0.05, ^**^P < 0.01, ^***^P < 0.001, *n* = 3, student *t*-test, means ± 95% CI).

To determine whether DYNC1I1 regulates the growth and migration of gastric cancer cell lines through the IL-6, we knocked down IL-6 in both HGC-27 and SGC-7901 cells and used qRT-PCR to verify the knockdown efficiency (Figure 6A). Knockdown of IL-6 significantly decreased the number of colonies formed by HGC-27 and SGC-7901 gastric cancer cells by approximately 35.7% (*P* < 0.05) and 45.7% (*P* < 0.05), respectively (Figure 6B). In addition, knockdown of IL-6 reduced the migration of HGC-27 and SGC-7901 gastric cancer cells by about 45.2 and 37.4%, respectively (*P* < 0.05, both cell lines) (Figure 6C). Then, after knocking down or overexpressing DYNC1I1, recombinant human IL-6 or IL-6 neutralizing antibody was added to detect changes in proliferation and migration. The addition of the IL-6 neutralizing antibody to DYNC1I1 expressing cells significantly inhibited proliferation (~60%). Conversely, the addition of IL-6 to DYNC1I1 knockdown cells reversed the inhibitory effect of DYNC1I1 knockdown on cell proliferation. Proliferation of the HGC-27 DYNC1I1 knockdown and SGC-7901 DYNC1I1 knockdown cells increased by about 50 and 60%, respectively, following the addition of recombinant human IL-6 (Figure 6D). Similarly, the use of IL-6 neutralizing antibodies can reverse the increase in proliferation caused by overexpression of DYNC1I1 (~30%). Treatment of HGC-27 DYNC1I1 knockdown and SGC-7901 DYNC1I1 knockdown cells with IL-6 increased cell migration by approximately 51 and 59.8%, respectively (Figure 6E). Treatment of MGC-803 DYNC1I1 overexpression cells with IL-6 neutralizing antibody decreased cell migration by ~60% (Figure 6F). Immunohistochemical staining revealed that IL-6 was downregulated in mouse subcutaneous tumors tissue and lung metastases tissue after DYNC1I1 knockdown (Figure 6G). Together, these results indicate that knockdown of DYNC1I1 suppressed cell growth and migration of gastric cancer cells by regulating the secretion of IL-6.

### DYNC1I1 Regulates IL-6 Expression by Promoting NF-κB Nuclear Transport

**Figure 7:**
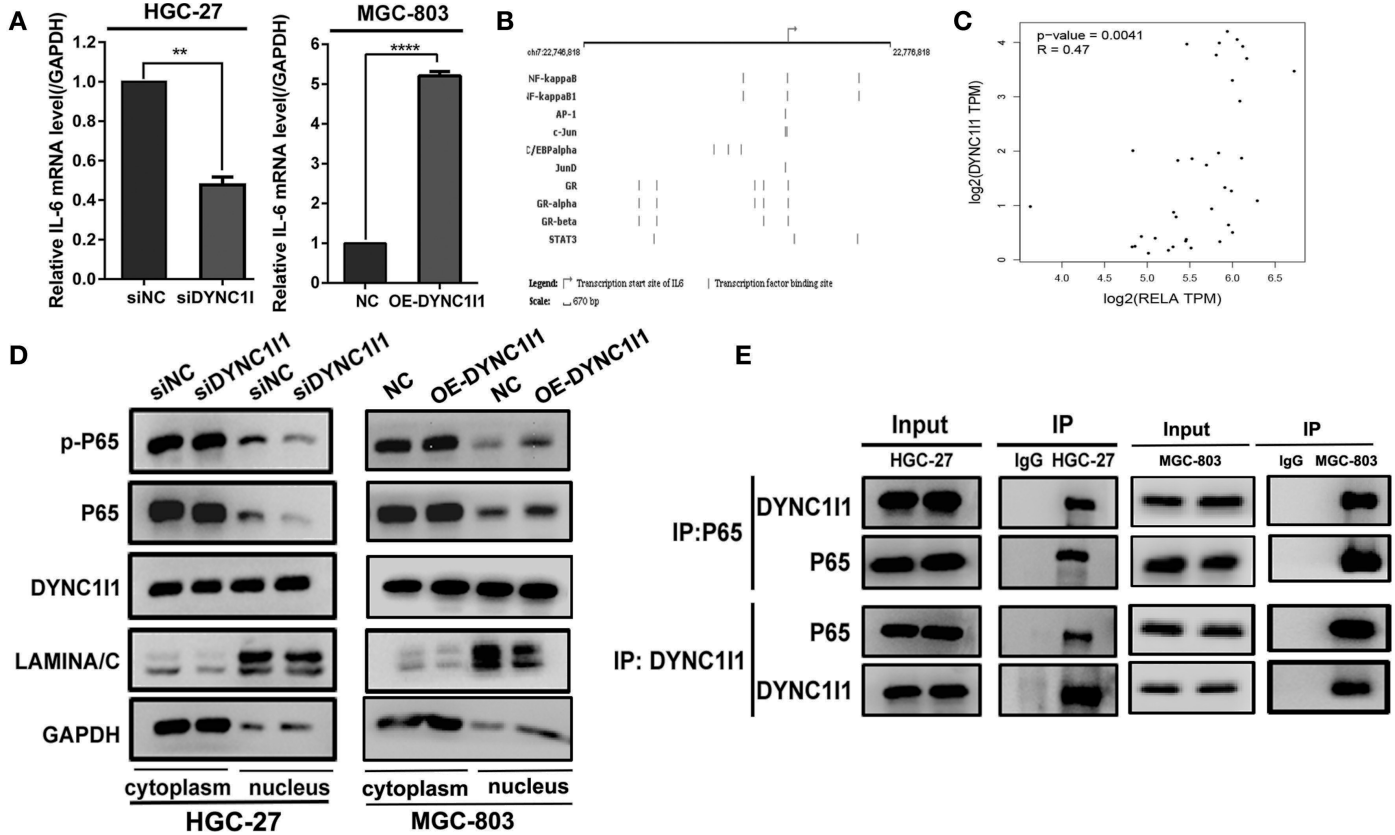
DYNC1I1 regulates IL-6 expression by promoting NF-KB nuclear transport. **(A)** RT-qPCR shows IL-6 transcription level after knocking down DYNC1I1 gene or overexpression DYNC1I1. **(B)** Website predicts IL-6 classical transcription factor (https://www.genecards.org/). **(C)** GEPIA website analysis the correlation between RELA(P65) and IL-6 in gastric cancer. **(D)** Nucleoplasm separation shows the distribution of P65 and P-P65 in the cytoplasm after knocking down DYNC1I1 or overexpression of DYNC1I1. **(E)** Immunoprecipitation shows the binding of P65 to DYNC1I1 in cells. (^**^P < 0.01, ^****^P < 0.0001, *n* = 3, student *t*-test, means ± 95% CI).

To explore the potential mechanisms by which DYNC1I1 regulates IL-6, we first used qRT-PCR to determine whether knockdown DYNC1I1 in HGC-27or overexpression of DYNC1I1 in MGC-803 cells could change IL-6 mRNA expression (Figure 7A). The result showed that IL-6 mRNA levels were significantly reduced in the DYNC1I1 knockdown cells compared to the control cells. Conversely, overexpression of DYNC1I1 in MGC-803 cells increased IL-6 mRNA level. We next predicted the IL-6-regulating transcription factors using GeneCard (GeneCards—Human Genes | Gene Database | Gene Search), and the top transcription factor was NF-κB (Figure 7B). A positive correlation between DYNC1I1 and RELA (Gene name of P65) was also predicted by GEPIA analysis (*P* < 0.05; Figure 7C). Using the nuclear and cytoplasmic protein extracts assay, we determined the distribution of NF-κB in the nucleus and cytoplasm of cells following the knockdown of DYNC1I1 (Figure 7D). DYNC1I1 knockdown caused a decrease in the amount of NF-κB/p65 and its phosphorylation level in the nucleus. Conversely, overexpression of DYNC1I1 in MGC-803 cells increased the amount of NF-κB/p65 and its phosphorylation level in the nucleus. Based on these results, DYNC1I1 might regulate IL-6 secretion through its regulation of NF-κB nuclear translocation. The distribution of DYNC1I1 itself in the gastric cancer cells following DYNC1I1 knockdown was detected. There was no significant change in the expression of DYNC1I1 in the nucleus, but its expression in the cytoplasm was downregulated. Because the role of DYNC1I1 in the cytoplasm is to transport cargos to the minus ends of the microtubules, co-immunoprecipitation was used to detect the binding of DYNC1I1 to P65 in HGC-27 and MGC-803 cells (Figure 7E). Western blot showed that DYNC1I1 could bind to P65. Taken together, these results showed that DYNC1I1 controls IL-6 expression levels by regulating NF-κB/p65 nuclear translocation in gastric cancer cells.

## Discussion

In the past, studies on the function of cytoplasmic dynein were mainly focused on its major constituent subunit, the heavy chain (DYNC1H1). DYNC1H1 is a driver gene in colorectal cancer progression (19). It is significantly upregulated in primary gallbladder carcinoma (PGC) and is a potential biomarker (20). Inactivating mutations of DYNC1H1 have been found to promote tumorigenesis in pancreatic cancer (21). The intermediate chain, DYNC1I1, has rarely been associated with tumors and its role in cancer remains controversial. In this study, we found that DYNC1I1 expression was upregulated in gastric cancer, and high DYNC1I1 expression was an independent prognostic factor for this disease. DYNC1I1 was associated with deeper infiltration depth (T stage), increased lymph node metastasis (N stage), and later staging (TNM stage) in gastric cancer. We speculate that DYNC1I1 promotes the advanced staging of gastric cancer (i.e., tumor progression), which results in poor patient prognosis.

To date, there have been several studies on the mechanisms by which cytoplasmic dynein regulates tumors. On the one hand, cytoplasmic dynein binds to microtubule-associated proteins to regulate the dynamic stability of microtubules. Microtubule dynamic stability is essential for tumor cell migration. For example, cytoplasmic dynein interacts with the microtubule-terminally related protein EB1 to promote fibrosarcoma cell migration through the activation of RhoA (10). Cytoplasmic dynein is also involved in the transport of transcription factors, growth factors, and other molecules from the cytoplasm to the nucleus. In neuroblastoma, cytoplasmic dynein plays a role in inhibiting tumor growth by promoting the transport of the tumor suppressor gene P53 into the nucleus (7). At the same time, in neurofibromatosis type 2 (NF2), cytoplasmic dynein transports the tumor suppressor protein Merlin into the nucleus. Merlin inhibits mitotic signaling by binding to the E3 ubiquitin ligase CRL4DCAF1 in the nucleus (22). In prostate cancer, cytoplasmic dynein promotes AR transport to the nucleus, resulting in prostate cancer progression (23).

Other studies show that cell cycle and DNA repair proteins or enzymes interact with cytoplasmic dynein (e.g., BRCA2, PKCε, PCNA, and MSH2) to promote cell cycle progression (24). At the same time, DYNC1I1 interacts with mammalian sphingosine kinase SK2 to sequester it away from the plasma membrane and promote tumor cell death in glioblastoma (6). DYNC1I1 can also bind to the intestinal cancer inhibitor Calgranulin B. But, it is not clear how to exert its inhibitory effect (14). Previously, Mikenberg et al. demonstrated that P53 and P65 must bind to the cytoplasmic dynein intermediate chain 1(DYNC1I1) in hippocampal neurons to be retrogradely transported along the axons of the neurons into the nucleus (25). P65 is a member of the NF-κB family of transcription factors that regulates various cancer-associated genes and plays a key role in tumorigenesis and development. If the microtubules required for dynein transport are disrupted, NF-κB cannot enter the nucleus. In this study, we demonstrated that P65 must bind to DYNC1I1 to gain entry into the nucleus in gastric cancer cells. Indeed, loss of DYNC1I1 impeded the translocation of ectopic P65 into the nucleus.

Although cytokines play essential roles in normal cellular processes, they can also contribute to tumor development. Previous reports have shown that cytoplasmic dynein is closely related to cytokines. LC8 is a structural subunit of cytoplasmic dynein. It inhibits RANKL-induced P-IκBα degradation and NFATc1 activation in osteoclast differentiation. NFATc1 is a determinant of osteoclastogenesis (26). In neurofibromatosis type 1, Cytoplasmic dynein can transport growth factors into the nucleus (27). In breast cancer, estrogen induces transcription and expression of DYNLC (28). In this study, we discovered that DYNC1I1 regulates the expression of the cytokine IL-6 by mediating the nuclear translocation of P65. IL-6 is a major cancer-promoting factor that activates a variety of pathways involved in tumor growth and tumor cell survival. Previous studies have demonstrated a relationship between IL-6 overexpression and increased cancer risk. Furthermore, High IL-6 expression is associated with poor prognosis in a variety of cancers.

**Figure 8:**
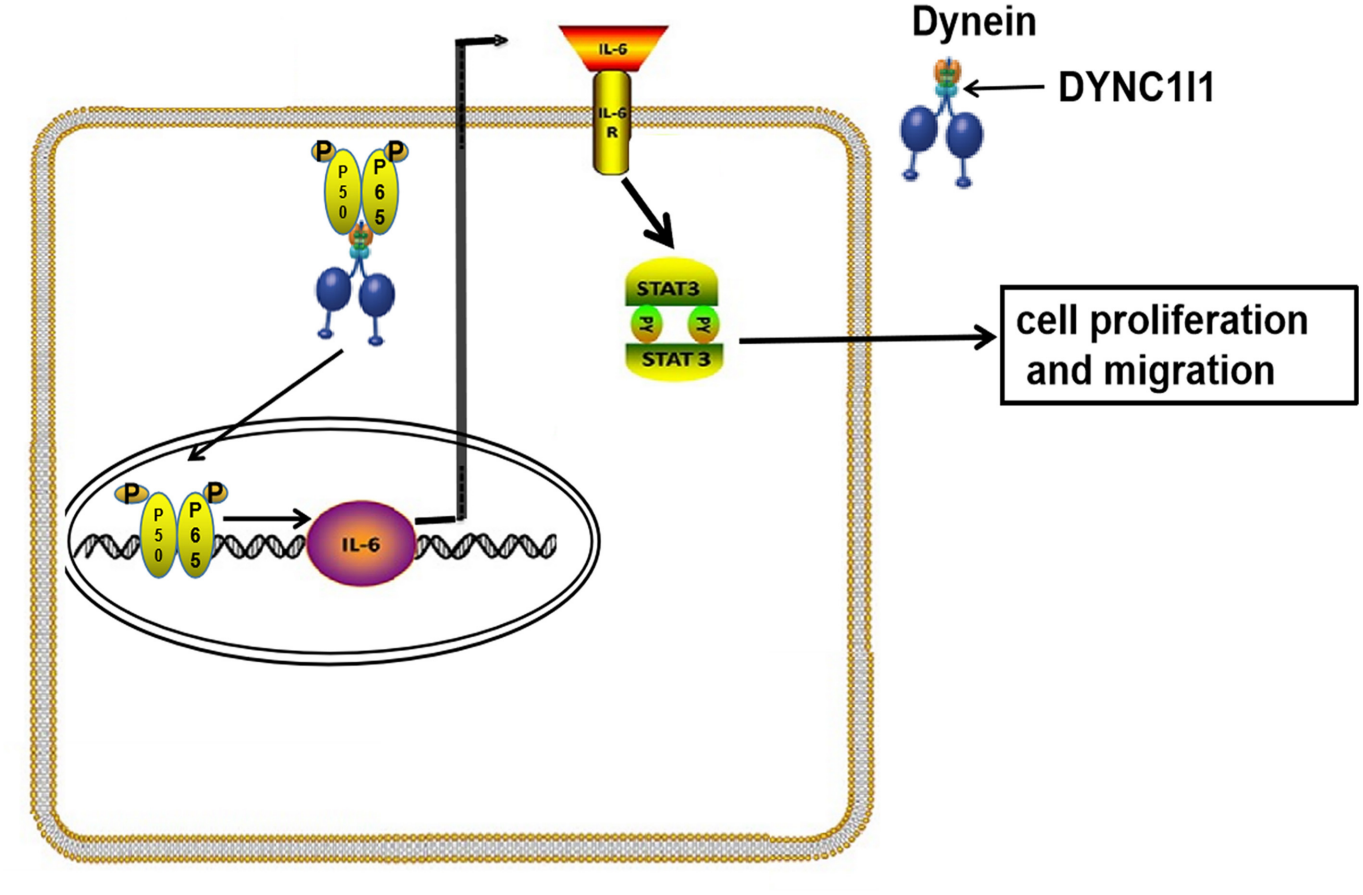
Working model for DYNC1I1in gastric cancer cells. In gastric cancer cells, DYNC1I1 acts as a binding subunit of cytoplasmic dynein. P65 is transported to the nucleus by cytoplasmic dynein in combination with DYNC1I1. P65 entering the nucleus to promote IL-6 transcription. Up-regulated IL-6 promotes proliferation and metastasis of gastric cancer cells by up-regulating phosphorylated stat3.

In summary, our study showed that gastric cancer patients with high DYNC1I1 expression had a poor prognosis. High levels of DYNC1I1 expression increased the proliferation and migration of gastric cancer cells. DYNC1I1 exerted its effects on gastric cancer cells by mediating the translocation of P65, which then upregulated IL-6 expression (The mechanism diagram is shown in Figure 8). This study revealed the role of DYNC1I1 in the biological behavior of gastric cancer cells, suggesting that DYNC1I1 may be a potential therapeutic target for the treatment of gastric cancer.

## Data Availability

Our experimental data is unpublished data in the past, and does not include data from other authors or other organizations. Part of the data comes from the public free-charged database. The source of database has been added to the article. The raw data supporting the conclusions of this manuscript will be made available by the authors, without undue reservation, to any qualified researcher.

## Ethics Statement

The animal study was approved by the Ethics Committee on Animal Care in China Medical University and all the experiments conform to the relevant regulatory standards. All research involving human participants were approved by the Ethics Committee of China Medical University. Written informed consents were obtained from all the participants in accordance with the Helsinki Declaration.

## Author Contributions

X-JQ and TW conceived and designed the study. ZL conducted data screening and analysis. L-BG did the experiments and analyzed results, and wrote the manuscript. JW, XX, and X-FC conducted experimental guidance. X-JQ and Y-PL revised the manuscript.

### Conflict of Interest Statement

The authors declare that the research was conducted in the absence of any commercial or financial relationships that could be construed as a potential conflict of interest.

## Supplementary Material

Supplementary Figure 1 The DYNC1I1 mRNA levels in GC tumors and normal tissues were analyzed using the Oncomine database.

Supplementary Table 1 Clinical pathologic parameters and DYNC1I1 expression level in primary gastric cancer.

## Abbreviations

GC: gastric cancer
DYNC1H1: Cytoplasmic dynein heavy chain
DYNC1I1: Cytoplasmic dynein intermediate chain
DYNLIC: Cytoplasmic dynein light intermediate chain
DYNLC: Cytoplasmic dynein light chain
Ki67: Nuclear related antigen Ki67
STAT3: Signal Transducer and Activator of Transcription3
IL-6: Interleukin 6.
