# Corrigendum: DYNC1I1 Promotes the Proliferation and Migration of Gastric Cancer by Up-Regulating IL-6 Expression

**DOI:** 10.3389/fonc.2022.819244

**Published:** 2022-02-24

**Authors:** Li-Bao Gong, Ti Wen, Zhi Li, Xing Xin, Xiao-Fang Che, Jin Wang, Yun-Peng Liu, Xiu-Juan Qu

**Affiliations:** ^1^ Department of Medical Oncology, the First Hospital of China Medical University, Shenyang, China; ^2^ Key Laboratory of Anticancer Drugs and Biotherapy of Liaoning Province, the First Hospital of China Medical University, Shenyang, China

**Keywords:** DYNC1I1, gastric cancer, proliferation, migration, IL-6, NF-κB nuclear translocation

In the original article, there were mistakes in **Figures 2F**, **5F** and **6E** as published. We identified minor errors which occurred during figure editing. The corrected **Figures 2**, **5** and **6** appear below.

**Figure 2:**
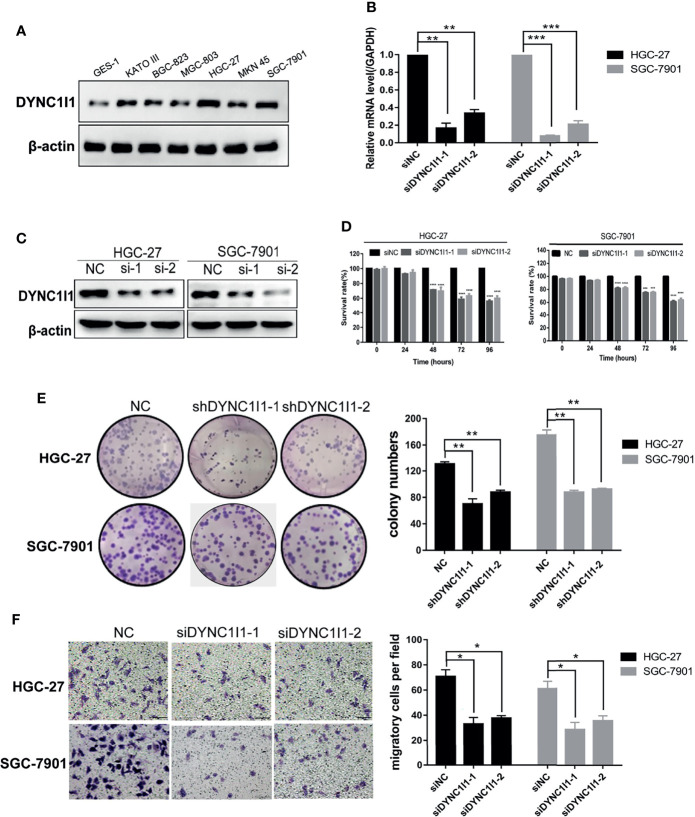
Knockdown of DYNC1I1 leads to suppression of gastric cancer progression and migration *in vitro*. **(A)** Western blot shows DYNC1I1 protein expression levels in normal gastric cells and different gastric cancer cells. **(B, C)** RT-qPCR and western blot show DYNC1I1 transcription level and protein expression after transient knockdown DYNC1I1 gene by using siRNAs for 48 h. **(D)** MTT shows cell viability of gastric cancer cells after knocking down DYNC1I1 for 0, 24, 48, 72, 96 h. **(E)** Colony formation shows gastric cancer cells form colony ability after knocking down DYNC1I1. **(F)** Transwell assay displays the change in migration of gastric cancer cells after knocking down DYNC1I1 or not (magnification × 200). (*P < 0.05, **P < 0.01,***P < 0.001, ****P < 0.0001, n = 3, student *t*-test, means ± 95% CI).

**Figure 5:**
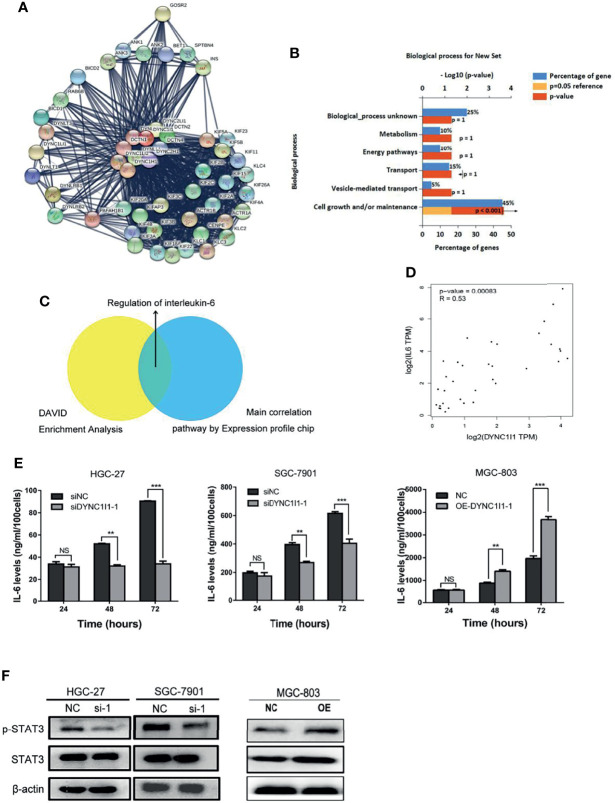
DYNC1I1 may function by regulating the IL-6 pathway. **(A)** Sting Website enrichment DYNC1I1 related genes and FunRich software enrichment related pathway. **(B)** FunRich software for Biological Pathway Enrichment Analysis. **(C)** Expression profile chip shows the main correlation pathway about DYNC1I1. **(D)** GEPIA website analysis the correlation between DYNC1I1 and IL-6 in gastric cancer. **(E)** Elisa shows the change of IL-6 expression after knocking down or overexpression DYNC1I1. **(F)** Western blot indicates the differential levels of STAT3, P-STAT3. β-actin was used as a loading control in Western blot. (**P < 0.01, ***P < 0.001, n = 3, NS, Not Statistically Significant student *t*-test, means ± 95% CI).

**Figure 6:**
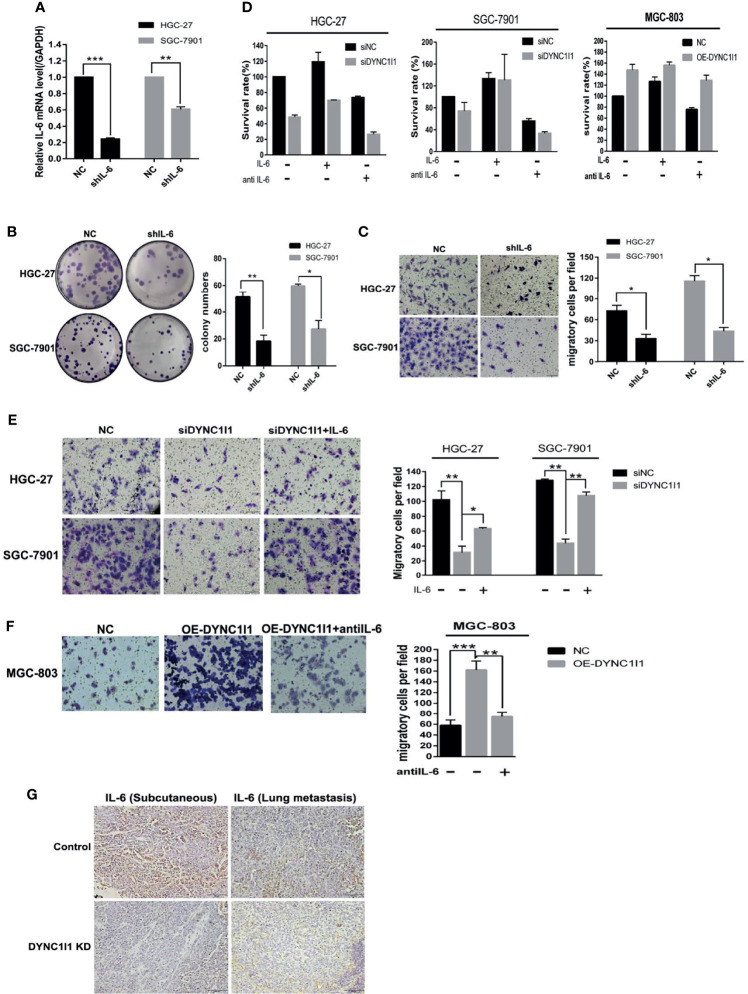
DYNC1I1 promotes proliferation and migration of gastric cancer cells through IL-6. **(A)** RT-qPCR shows IL-6 transcription levels after knockdown IL-6 gene by using shRNA for 48 h. **(B)** Colony formation shows gastric cancer cells form colony ability after knocking down IL-6. **(C)** Transwell assays displays the change in migration of gastric cancer cells after knocking down IL-6 or not. **(D)** MTT shows proliferation ability of gastric cancer cells after adding IL-6 or IL-6 neutralizing antibody after knocking down or overexpression DYNC1I1. Add IL-6 or IL-6 neutralizing antibody 48 h after knocking down DYNC1I1. **(E)** Transwell assays shows the change in migration of gastric cancer cells after adding IL-6 after knocking down DYNC1I1. **(F)** Transwell assays shows the change in migration of gastric cancer cells after adding IL-6 neutralizing antibodies after overexpression DYNC1I1. (magnification ×200) **(G)** Immunohistochemical detection of IL-6 expression in subcutaneous and lung metastatic tumors of mice tumor tissue(magnification × 100). (*P < 0.05, **P < 0.01, ***P < 0.001, n = 3, student *t*-test, means ± 95% CI).

The authors apologize for this error and state that this does not change the scientific conclusions of the article in any way. The original article has been updated.

## Publisher’s Note

All claims expressed in this article are solely those of the authors and do not necessarily represent those of their affiliated organizations, or those of the publisher, the editors and the reviewers. Any product that may be evaluated in this article, or claim that may be made by its manufacturer, is not guaranteed or endorsed by the publisher.

