# Supplementary figures and images for: DYNC1I1 Promotes the Proliferation and Migration of Gastric Cancer by Up-Regulating IL-6 Expression
Source: Front Oncol. 2019 Jun 12;9:491. doi: 10.3389/fonc.2019.00491 (PMC6582752; doi:10.3389/fonc.2019.00491)

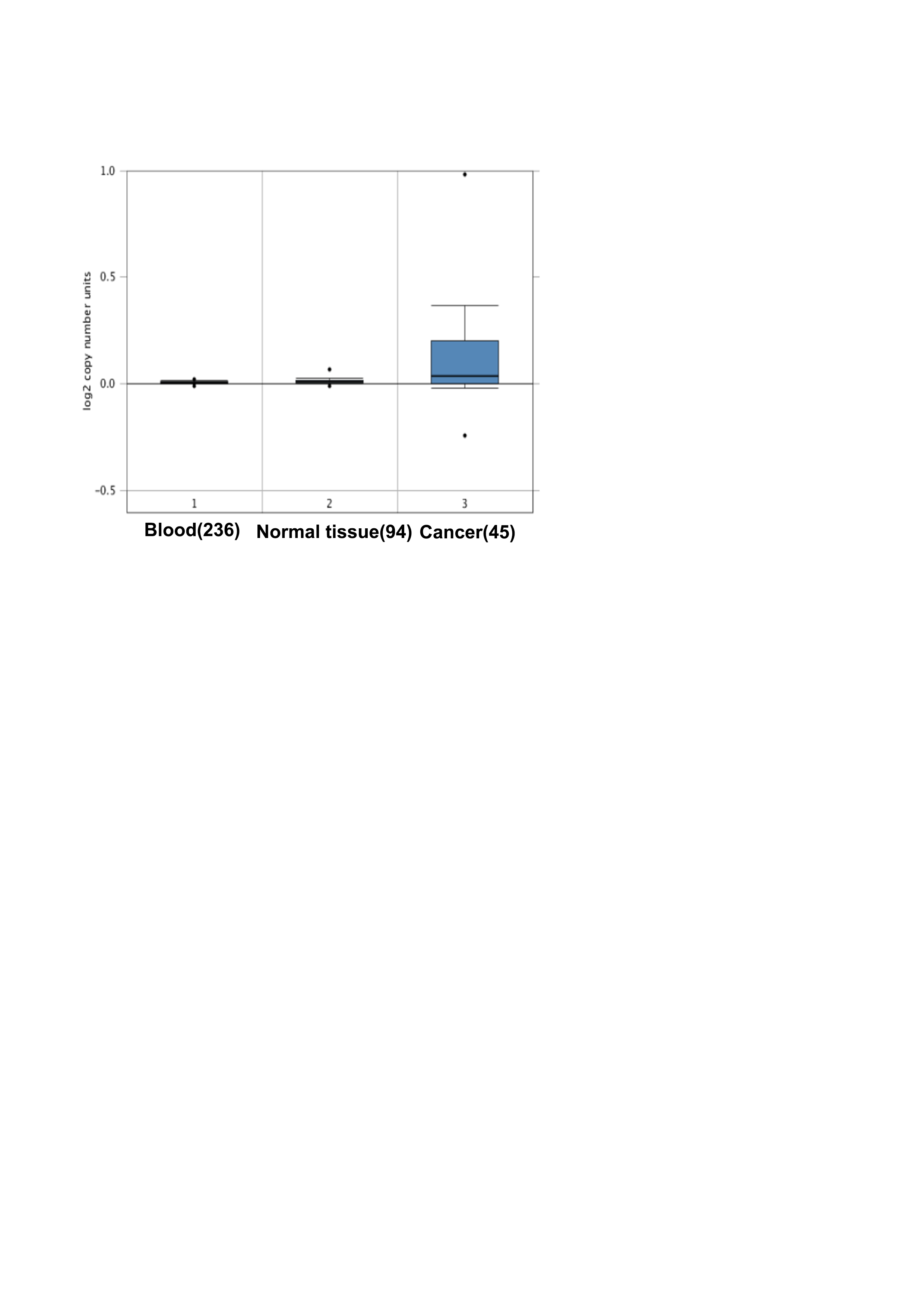

Supplement: Supplementary Figure 1 — The DYNC1I1 mRNA levels in GC tumors and normal tissues were analyzed using the Oncomine database. [file Image_1.TIF]
