# Supplementary material for: DYNC1I1 Promotes the Proliferation and Migration of Gastric Cancer by Up-Regulating IL-6 Expression
Source: Front Oncol. 2019 Jun 12;9:491. doi: 10.3389/fonc.2019.00491 (PMC6582752; doi:10.3389/fonc.2019.00491)
Supplement: Supplementary Table 1 — Clinical pathologic parameters and DYNC1I1 expression level in primary gastric cancer. [file Table_1.docx]

Supplementary Material

Table S1:Clinical pathologic parameters and DYNC1I1 expression level in primary gastric cancer

| **Patient and tumor characteristics** | | | | **DYNC1I1 expression level*** | |
| --- | --- | --- | --- | --- | --- |
| No. | Age, yr/Sex | Volume, cm | pTNM | Normal | Tumor |
| 1 | 56/F | 2X3X0.3 | IA | 2 | 6 |
| 2 | 42/M | 3.2X4X0.2 | IA | 5 | 8 |
| 3 | 57/F | 1.2X1.5X0.2 | IA | 4 | 2 |
| 4 | 62/M | 4X3X0.2 | IA | 2 | 6 |
| 5 | 76/F | 1X1.2X1 | IA | 2 | 6 |
| 6 | 49/M | 2X3.3X0.3 | IA | 3 | 4 |
| 7 | 64/M | 2.5X2X0.3 | IA | 6 | 5 |
| 8 | 60/M | 2.8X2X0.4 | IA | 5 | 8 |
| 9 | 51/F | 2.3X2.4X0.8 | IA | 4 | 2 |
| 10 | 60/M | 6X4X0.3 | IA | 6 | 4 |
| 11 | 61/F | 2.9X2.5X0.2 | IA | 5 | 2 |
| 12 | 73/F | 1X2.3X0.3 | IA | 4 | 4 |
| 13 | 54/M | 4.8X5.5X0.5 | IA | 2 | 7 |
| 14 | 64/M | 1X0.6X0.2 | IA | 5 | 6 |
| 15 | 67/M | 2.5X3X0.2 | IA | 5 | 2 |
| 16 | 62/M | 2X2.8X0.8 | IB | 3 | 4 |
| 17 | 73/M | 1.6X1X0.4 | IB | 2 | 8 |
| 18 | 55/M | 4.5X5.5X1.7 | IIB | 5 | 4 |
| 19 | 77/F | 6X5X1.2 | IIB | 2 | 6 |
| 20 | 76/F | 8.5X7.5X0.9 | IIB | 2 | 6 |
| 21 | 63/M | 2.5X3X1 | IIB | 2 | 4 |
| 22 | 42/M | 7X6.5X3 | IIIA | 2 | 2 |
| 23 | 47/M | 4.6X5X1.1 | IIIA | 2 | 6 |
| 24 | 43/F | 4.5X4X0.8 | IIIA | 2 | 6 |
| 25 | 46/F | 2.8X3X0.5 | IIIB | 4 | 8 |
| 26 | 57/F | 6.7X6X0.6 | IIIB | 5 | 4 |
| 27 | 27/F | 6X6X1 | IIIB | 5 | 6 |
| 28 | 70/M | 7X5X1.2 | IIIC | 3 | 5 |
| 29 | 74/M | 4X4X2.7 | IIIC | 2 | 3 |
| 30 | 60/F | 2.3X3.5X1.2 | IIIC | 3 | 4 |
| *DYNC1I1 expression levels in cancerous and matched noncancerous tissues were analyzed by IHC. | | | | | |
